# Eigenkapitalpuffer im Abschwung wirksam?

**DOI:** 10.1007/s10273-021-2875-8

**Published:** 2021-03-17

**Authors:** Franziska Bremus, Lukas Menkhoff

**Affiliations:** 1 Abteilung Makroökonomie, Deutsches Institut für Wirtschaftsforschung, Mohrenstraße 58, 10117 Berlin, Deutschland; 2 Abteilung Weltwirtschaft, Deutsches Institut für Wirtschaftsforschung, Mohrenstraße 58, 10117 Berlin, Deutschland

**Keywords:** G21, G28

## Abstract

Der antizyklische Kapitalpuffer und der Kapitalerhaltungspuffer wurden für die Verstetigung der Kreditvergabe sowie eine höhere Resilienz des Finanzsystems geschaffen. Fraglich bleibt, wie gut diese Puffer speziell im Abschwung in der Praxis von den Finanzinstituten genutzt werden. Dem Signalisierungsproblem zufolge könnte eine sinkende Eigenkapitalquote als positives (Fähigkeit zu Neugeschäften) aber auch negatives Zeichen (geringere Stabilität) gedeutet werden. Hinzu kommt das Unsicherheitsproblem, bei dem Kreditinstitute freigesetztes Eigenkapital in unsicheren Zeiten nicht für zusätzliches Kreditgeschäft nutzen.

## Abstract (en)

Additional “capital buffers”, such as the countercyclical capital buffer, were introduced after the 2008/09 crisis. These buffers were requested in addition to banks’ minimum capital requirements. They should be built up during good times and can be used during bad times. While buffer requirements were reduced quickly in the spring of 2020, financial institutions have been reluctant to use the additional leeway as they may fear market stigma effects if capital ratios fall and they might abstain from new loans due to high uncertainty. The article argues in favor of a more simple and transparent organisation of macroprudential regulation as measures to raise the effectiveness of capital buffers, especially during a downturn.

Zur Sicherstellung der Finanzstabilität ist seit der letzten globalen Finanzkrise von 2008/2009 das Instrumentarium der finanzaufsichtlichen Maßnahmen ausgebaut worden. Insbesondere wurde bei der Festlegung des notwendigen Eigenkapitals von Kreditinstituten das Mindestniveau und dessen Qualität deutlich erhöht (Bremus und Lambert, 2014). Ferner sind zusätzliche „Puffer“ geschaffen worden, darunter der antizyklische Kapitalpuffer und der Kapitalerhaltungspuffer. Eigenkapitalpuffer sind über das permanente Minimum hinausgehende Anforderungen an die Eigenkapitalausstattung, die je nach wirtschaftlicher Situation gelockert oder verschärft bzw. genutzt werden können. Sie sollen entweder in guten Zeiten, wenn die Kreditinstitute dies leisten können, aufgebaut und in schlechten Zeiten wieder abgebaut werden, oder sie können temporär genutzt werden. Mit diesem antizyklischen Auf und Ab soll zu einer Verstetigung der Kreditvergabe und zur Resilienz des Finanzsystems beigetragen werden (Shim, 2013).

So überzeugend diese Überlegung theoretisch ist, so unklar ist derzeit, inwieweit sie sich in der Praxis umsetzen lässt. Beim Aufbau des antizyklischen Kapitalpuffers sind die Länder in Europa unterschiedlich vorgegangen. Einige handelten entschlossener als andere, Deutschland agierte eher zögerlich (Gischer et al., 2019). Im Vergleich dazu hat die Rücknahme der antizyklischen Pufferanforderung im Frühjahr 2020 sehr zügig funktioniert. Nun stellen sich möglicherweise zwei andere Probleme, die von den Kreditinstituten ausgehen:

Werden *erstens* Puffer freigegeben, bieten sie den betreffenden Kreditinstituten „freies“ Eigenkapital. Ziel dieser Lockerung der vorgeschriebenen Eigenkapitalquoten ist es, mehr Spielraum für zusätzliches Kreditgeschäft oder eine Absorption von Verlusten zu ermöglichen. Sinkt die Eigenkapitalquote eines Kreditinstituts nach der regulatorischen Lockerung, können die Märkte dies als gutes (Fähigkeit zu Neugeschäft), aber auch schlechtes Zeichen (geringere Stabilität) werten.[1] Die Kreditinstitute können deshalb davor zurückschrecken, die gewährten Puffer zu nutzen. *Zweitens* müssen die Institute über das frei gewordene Eigenkapital unter unsicheren Umständen entscheiden. Die Lockerung der Eigenmittelanforderungen erfolgt gerade, weil die gesamtwirtschaftliche Lage schwierig ist. Wenn einerseits der künftige Abschreibungsbedarf und die Entwicklung der Kreditnachfrage schwerer einzuschätzen und andererseits die Bedingungen für Eigenkapitalbildung möglicherweise erschwert sind, ist es riskant, freies Eigenkapital zu binden. Darüber hinaus besteht Unsicherheit über das Auslaufen der fiskalischen Stützungsmaßnahmen.

Auch wenn es im Euroraum und besonders in Deutschland bislang keine Anzeichen für eine Kreditklemme gibt, bestehen Hinweise darauf, dass die beiden Probleme eine Rolle

1 Die Lockerung eines Eigenkapitalpuffers lässt das Eigenkapital und die Eigenkapitalquote zunächst unverändert. Sie setzt nur vormals gebundenes Eigenkapital frei. Wenn neues Geschäft gemacht wird, das die Risikoaktiva erhöht, sinkt die Eigenkapitalquote, wenngleich der Eigenkapitalspielraum der Kreditinstitute so lange erhöht bleibt, bis sie entweder den gesamten neu geschaffenen Spielraum für Neugeschäft verwenden und/oder zusätzlicher Abschreibungsbedarf das Eigenkapital reduziert.

* Der/die Autor:in danken Claudia Buch und Mario Jovanovic für sehr hilfreiche Kommentare.

**Dr. Franziska Bremus** ist Forschungsgruppenleiterin in der Abteilung Makroökonomie am DIW Berlin.

**Prof. Dr. Lukas Menkhoff** lehrt VWL an der Humboldt-Universität zu Berlin und leitet die Abteilung Weltwirtschaft am DIW Berlin.

spielen (EZB, 2020, 104 ff): Kreditinstitute möchten negative Signale vermeiden, dass sie entweder freiwerdendes Eigenkapital benötigen, um künftige Kreditausfälle zu decken, oder aber bei fallenden Kapitalquoten in Zukunft weniger handlungsfähig sein könnten. So gibt es Hinweise darauf, dass Kreditinstitute, die mit ihren Eigenmitteln nahe an den kombinierten Pufferanforderungen liegen, mit einem Abbau riskanter Wertpapiere und mit der Verringerung von Risikogewichten reagiert haben – allerdings bei gleichzeitiger Ausweitung der Kreditvergabe (EZB 2020, Abb. 5.1). Grundsätzlich können sich fallende Kapitalquoten in schlechteren Ratings der Kreditinstitute und höheren Finanzierungskosten niederschlagen oder sogar zu vorübergehenden Beschränkungen beim Zugang zu Anleihemärkten führen (Andreeva et al., 2020). Diese Sorgen vor ungünstigen Marktreaktionen könnten die Institute davon abhalten, ihre Kreditvergabe weniger prozyklisch zu gestalten. Stattdessen könnten sie bei steigendem Abschreibungsbedarf über eine Verknappung des Kreditangebots die Eigenkapitalquoten stabilisieren.

Im Zuge der finanziellen Globalisierung ist seit den 1980er Jahren die Regulierung von Kreditinstituten international vereinheitlicht worden. Die ersten Rahmenwerke haben vor allem versucht, übliche nationale Regulierungswerke zu harmonisieren, um einen fairen Wettbewerb zu fördern. Erst infolge der Finanz- und Wirtschaftskrise von 2008/2009 ist es zu einer deutlichen Erhöhung der Anforderungen gekommen. Es war offensichtlich, wie dünn die Eigenkapitalausstattung der Kreditinstitute war und damit auch die Fähigkeit, unerwartete Schocks selbst abzufedern. Stattdessen haben in vielen Ländern staatliche Einrichtungen die Kreditinstitute stabilisiert. Um diese Unterstützung zukünftig weniger wahrscheinlich zu machen und in ihrem Umfang geringer zu halten, sind unter anderem die Eigenkapitalanforderungen heraufgesetzt worden. Größenordnungsmäßig sind die gewichteten regulatorischen Kapitalquoten heute doppelt so hoch wie vor der Krise 2008/2009 (vgl. Abbildung 1).[2] Zweifellos ist der Finanzsektor dadurch stabiler geworden. Dennoch stellt sich die Frage, ob das aufgebaute Eigenkapital allein groß genug ist, um potenziell anfallende Verluste zu decken und auch längerfristig einen Rückgriff auf staatliche Stützungsmaßnahmen zu verhindern (Admati und Hellwig, 2013).

## Die Idee der Eigenkapitalpuffer

Die makroprudenzielle Regulierung stellt zusätzlich Instrumente bereit, um den Finanzsektor und die Kreditinstitute widerstandsfähiger zu machen (z. B. Galati und Moessner, 2013). Ein zentraler Bestandteil des makroprudenziellen

Abbildung 1

**Kernkapitalquoten seit der globalen Finanzkrise**

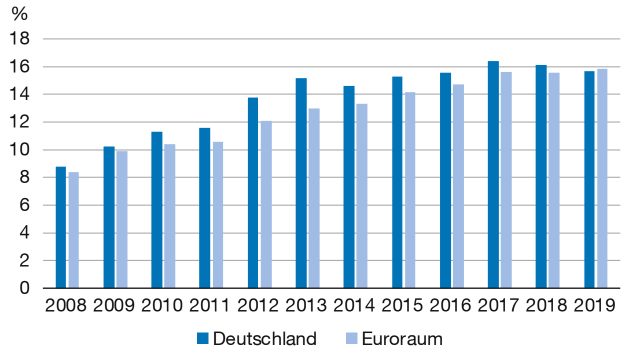

Quelle: Europäische Zentralbank, Supervisory and Prudential Statistics, Consolidated Banking Data.

Maßnahmenkatalogs sind Eigenkapitalpuffer. Mit der Umsetzung des Basel-III-Abkommens sind zwei Puffer eingeführt worden, die grundsätzlich alle europäischen Kreditinstitute betreffen, unabhängig von deren Größe oder Geschäftsmodell: zum einen der Kapitalerhaltungspuffer, zum anderen der antizyklische Kapitalpuffer.[3] Der Kapitalerhaltungspuffer von 2,5 % der risikogewichteten Aktiva kommt zur Mindesteigenkapitalquote von 8 % hinzu, ist also beträchtlich. Sofern ein Institut dieses Eigenkapital nicht vorweisen kann, unterliegt es Restriktionen bei Dividendenausschüttungen, Boni oder Aktienrückkäufen, die darauf hinwirken, Eigenkapital aufzubauen. Damit sichert dieser Puffer die Handlungsfähigkeit des Instituts in schwierigen Zeiten.

Aufgrund der Corona-Pandemie gewähren die europäischen und deutschen Aufsichtsbehörden den Kreditinstituten Kapitalerleichterungen, indem sie diese Puffer freigeben bzw. nutzbar machen. Die EZB hat den Kreditinstituten seit dem Frühjahr 2020 Kapitalerleichterungen gewährt. So dürfen die Kreditinstitute z. B. entsprechend der wirtschaftlichen Entwicklung unterhalb des Kapitalerhaltungspuffers operieren, um den Spielraum für die Kreditvergabe zu erhöhen oder gegebenenfalls Verluste zu absorbieren. Gleichzeitig haben sowohl die EZB als auch die Bundesanstalt für Finanzdienstleistungsaufsicht (BaFin) nur Empfehlungen gegen eine Ausschüttung von Dividenden, Gewinnen und Boni ausgesprochen (BaFin, 2020).

Anders ist der antizyklische Kapitalpuffer konzeptioniert. Dieser gilt ebenfalls für alle Kreditinstitute, aber spezifisch je Land, und kann von den zuständigen Gremien (wie der BaFin) zwischen 0 und (in der Regel) 2,5 % der risikogewichteten Aktiva festgelegt werden. Dieser Puffer soll bei starkem Kre-

2 Die nicht-risikogewichtete Quote vom gesamten Eigenkapital zur Bilanzsumme der Kreditinstitute im Euroraum lag mit rund 6,5 % zuletzt weiterhin deutlich unterhalb der gewichteten Eigenkapitalquote. 2008 lag die einfache Kapitalquote im Euroraum bei rund 4 %.

3 Daneben gibt es weitere Eigenkapitalpuffer für Kreditinstitute, die für den Finanzsektor systemrelevant sind. Diese Puffer werden in Abhängigkeit von Indikatoren zu Größe, Verflochtenheit und Komplexität festgelegt und jährlich angepasst (Deutsche Bundesbank).

ditwachstum in Relation zur realwirtschaftlichen Entwicklung aufgebaut werden, um eine übermäßige Ausweitung der Kreditvergabe abzubremsen und das Finanzsystem resilienter zu machen. In schlechten Zeiten können diese Puffer abgeschmolzen werden, um einer krisenbedingten prozyklischen Restriktion des Kreditangebots entgegenzuwirken, welche die realwirtschaftliche Entwicklung zusätzlich bremsen würde (Buch et al., 2016). Nachdem die BaFin den antizyklischen Kapitalpuffer für die deutschen Kreditinstitute im Juli 2019 von null auf 0,25 % erhöht hatte, wurde diese zusätzliche Kapitalanforderung im Zuge der Corona-Pandemie im April 2020 wieder zurückgenommen. Da die Kreditinstitute diese Quote erst im Juli 2020 hätten umsetzen müssen, dürfte die Rücknahme dieser Pufferanforderung in Deutschland also nicht voll wirksam gewesen sein. Insgesamt variierte sowohl die Nutzung als auch die Ausgestaltung dieses relativ neuen makroprudenziellen Regulierungsinstruments vor der Corona-Pandemie zwischen den Ländern, während fast alle europäischen Länder seit dem Frühjahr 2020 den antizyklischen Kapitalpuffer auf null gesetzt haben (Arbatli-Saxegaard und Muneer, 2020; EZB). Im Euroraum sorgte dies seit Beginn der Pandemie damit für moderate Kapitalerleichterungen von etwa 20 Mrd. Euro (EZB, 2020, 103).

## Das Signalisierungsproblem

Das Ziel solcher Regulierungsanpassungen ist, in wirtschaftlich guten Zeiten Kapitalpuffer aufzubauen, die dann in schlechten Zeiten aufgezehrt werden können. In der aktuellen Situation deutet sich aber an, dass die Puffer nicht immer im gewünschten Maße genutzt werden. Als ein Grund hierfür wird die Angst vor Marktreaktionen genannt (Andreeva et al., 2020; EZB, 2020). Ein Beispiel ist die Entwicklung der Liquiditätsdeckungsquote, also von Liquiditätspuffern, die ebenfalls im Zuge von Basel III eingeführt wurden.[4] In dieser Kennzahl zeigten sich im zweiten Quartal 2020 Effekte der Pandemie auf den Bankensektor, die neben den geldpolitischen Liquiditätsmaßnahmen auch mit einem Stigmatisierungsproblem zusammenhängen könnten. Denn obwohl die EZB unterstrichen hatte, dass Kreditinstitute temporär auch unterhalb der regulatorisch vorgeschriebenen Grenze der Liquiditätsdeckungsquote von 100 % liegen dürfen, war im Euroraum im Frühjahr 2020 statt eines Rückgangs eine deutliche Zunahme auf durchschnittlich 210 % zu beobachten (vgl. Abbildung 2). In Deutschland nahm die Liquiditätsquote im zweiten Quartal 2020 auch zu, aber in geringerem Umfang.

Mittel- und längerfristig könnte sich ein ähnliches Muster bei der Nutzung der Kapitalpuffer abzeichnen. Wird die-

4 Die Liquiditätsdeckungsquote soll gewährleisten, dass Kreditinstitute unter Stress über genügend liquide, hochwertige Aktiva verfügen. Die Quote berechnet sich aus dem Bestand an verfügbaren Zahlungsmitteln in Relation zu den abrufbaren Zahlungsverpflichtungen der nächsten 30 Tage und muss in normalen Zeiten mindestens 100 % betragen.

Abbildung 2
**Liquiditätsdeckungsquoten**

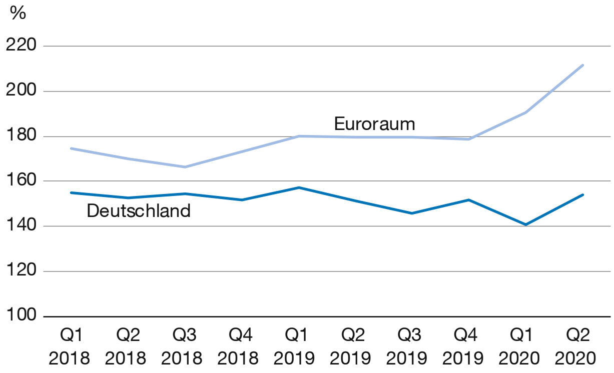

Quelle: Europäische Zentralbank, Supervisory Banking Statistics.

ses „freigesetzte“ Eigenkapital für zusätzliches Geschäft genutzt, sollte die Eigenkapitalquote – auf hohem Niveau – sinken. Tatsächlich ist bis zur Jahresmitte 2020 noch keine signifikante Veränderung der harten Kernkapitalquote (CET1) zu erkennen. Wenn es in den kommenden Quartalen zu einer höheren Zahl an Insolvenzen kommen sollte (Demary und Hüther, 2020; Gropp et al., 2020; Clemens et al., 2020) ist also nicht klar, inwiefern Kreditinstitute die freigegebenen Kapitalpuffer nutzen und ihre Eigenkapitalquoten reduzieren, um weiterhin Kredite zu vergeben oder Verluste aufzufangen. Durch einen Rückgang der Eigenkapitalquote können Kreditinstitute vom Markt stigmatisiert werden.

## Das Unsicherheitsproblem

Während das Signalisierungsproblem auf die Sichtweise der Märkte abzielt, betrifft das Unsicherheitsproblem vor allem das Management des jeweiligen Kreditinstituts. Die Lockerung von Eigenkapitalanforderungen erfolgt im Rahmen der makroprudenziellen Steuerung immer in gesamtwirtschaftlich schwierigen Zeiten, in denen das Management nicht genau absehen kann, welche Belastungen auf das Institut zukommen: Im Abschwung (und meist noch im beginnenden Aufschwung) steigt der Abschreibungsbedarf, gleichzeitig sind die Gewinne niedrig und die Möglichkeiten, das Eigenkapital zu erhöhen, schlecht. Je tiefer und andauernder ein Abschwung dauert, desto gravierender werden diese Schwierigkeiten. So ist verständlich, dass freigesetztes Eigenkapital in solchen Phasen allein aus Sicht des Managements nicht unbedingt für zusätzliches Kreditgeschäft genutzt wird. Während also manches dafür spricht, dass freigesetzte bzw. nutzbare Eigenkapitalpuffer aufgrund der hohen Unsicherheit in solchen Phasen nicht für zusätzliches Geschäft verwendet werden, ist aber auch klar, dass die Kapitalerleichterungen einen möglichen Druck mildern, Kredite in Stresssituationen zu verknappen.

Bislang ist es auf dem Kreditmarkt nur vorübergehend zu Anspannungen gekommen. Die Kreditnachfrage ist laut dem Bank Lending Survey der EZB nach einem starken Anstieg im ersten und zweiten Quartal 2020 im dritten Quartal wieder merklich zurückgegangen (vgl. Abbildung 3). So lag der Anteil der Kreditinstitute, die eine gestiegene Nachfrage nach Kreditlinien und Liquidität meldeten abzüglich dem Anteil derer, die einen Nachfragerückgang verzeichneten (Nettoanteil), in der ersten Phase der Corona-Pandemie im Euroraum bei bis zu 60 %. Zwischen Juli und September ging der Nettoanteil auf -4 % zurück – der Anteil von Instituten mit rückläufiger Kreditnachfrage überstieg also den Anteil derer, die eine gestiegene Nachfrage meldeten. Für das Schlussquartal erwarteten die befragten Kreditinstitute im Euroraum dagegen wieder eine etwas zunehmende Nachfrage. Während die Kreditnachfrage für Anlageinvestitionen einbrach, trug die deutlich gestiegene Nachfrage nach Finanzierung für Lagerhaltung und Umlaufkapital positiv zur gesamten Kreditnachfrage bei. In Deutschland war ein ähnliches Muster zu beobachten. Auch hier ging die Kreditnachfrage im dritten Quartal 2020 zurück und dürfte gemäß der Erwartung der Kreditinstitute zum Jahresende wieder leicht zugenommen haben. Hier berichtete aber nur ein Nettoanteil von 10 % bis 20 % der befragten Kreditinstitute einen Rückgang der Kreditnachfrage für Anlageinvestitionen, während dieser Wert im Euroraum bei 20 % bis 40 % lag.

Die Kreditvergabestandards blieben zu Beginn der Corona-Pandemie im Euroraum nahezu unverändert. Allerdings meldete ein Nettoanteil der befragten Kreditinstitute von knapp 20 % im dritten Quartal 2020 eine restriktivere Kreditvergabe als im Vorquartal, was auch im vierten Quartal so geblieben sein dürfte (vgl. Abbildung 4). Wichtige Gründe für diese Straffung sind die wirtschaftliche Situation sowie die unsicheren Aussichten, welche die Risikoaversion der Kreditinstitute erhöhen. In Deutschland berichtete zwar ein abnehmender Anteil der Institute eine restriktivere Kreditvergabe, dennoch blieb der Nettoanteil der Institute mit strengeren Kreditvergabestandards bis zuletzt positiv. Für das Schlussquartal 2020 rechnen die Kreditinstitute hierzulande allerdings vermehrt mit einer Lockerung der Standards. Zusammenfassend war Ende 2020 also eine gestiegene Kreditnachfrage in Kombination mit einer Eintrübung der Kreditkonditionen zu beobachten, was die Situation der Unternehmen verschlechtert – in Deutschland weniger als im Euroraum. Insofern ist die Reduktion der Eigenkapitalpuffer eine grundsätzlich angemessene Gegenmaßnahme, um einer möglichen krisenverschärfenden Kreditklemme entgegenzuwirken.

## Grenzen makroprudenzieller Politik

Die Frage, ob Eigenkapitalpuffer im Abschwung möglicherweise nicht so aktiv genutzt werden, wie konzeptionell gewünscht, kann zurzeit nicht abschließend bewertet werden, dennoch gibt es einen umfassenden Diskussionszusammenhang zu möglichen Problemen makroprudenzieller Politik. Demnach steht die makroprudenzielle Regulierung im Widerstreit zwischen aktiven Regulierenden (mit gesamtwirtschaftlichem Interesse) und bremsenden Regulierten (mit einzelwirtschaftlichem Interesse) (Bengtsson, 2020; Goodhart, 2015). Es wird argumentiert, dass letztere aufgrund der Kleinteiligkeit und des hohen technischen Verständnisses auf dieses wirtschaftspolitische Instrument relativ großen Einfluss nehmen können (z. B. Kranke und Yarrow, 2019). Aus dieser Perspektive mag man es als Extremposition bestreiten, dass makroprudenzielle Politik vom Ansatz her ein gutes wirtschaftspolitisches Instrument ist. Man kann die Kritik auch konstruktiv wenden und daraus die Anforderung ableiten, dass solch eine Politik einfach ausgestaltet sein sollte, um sie weniger anfällig für selektive Abschwächungen zu machen.

Als Vergleich dient die Geldpolitik, die genereller als makroprudenzielle Regulierung ausgestaltet ist und auch genereller angewendet wird. Dies kann dazu beitragen, sich besser aus dem Streit verschiedener Interessengruppen herauszuhalten.

Abbildung 3
**Kreditnachfrage der nicht-finanziellen Unternehmen**

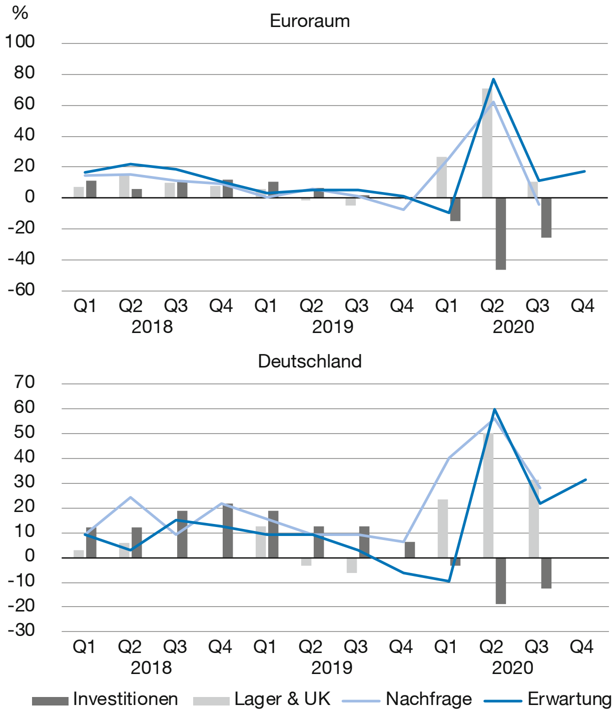

Anmerkung: Anteil der Kreditinstitute, die gestiegene Nachfrage im vergangenen Quartal berichten abzüglich der, die gefallene Nachfrage berichten. Investitionen = Auswirkung der Anlageinvestitionen (Nettoanteil Unternehmen, die mehr Kredite für Anlageinvestitionen nachgefragt haben), Lager & UK = Auswirkung von Lagerhaltung und kurzfristigem Umlaufkapital.

Quelle: Europäische Zentralbank, Bank Lending Survey, Kreditnachfrage.

Abbildung 4

**Kreditvergabestandards und ausgewählte Einflussfaktoren**

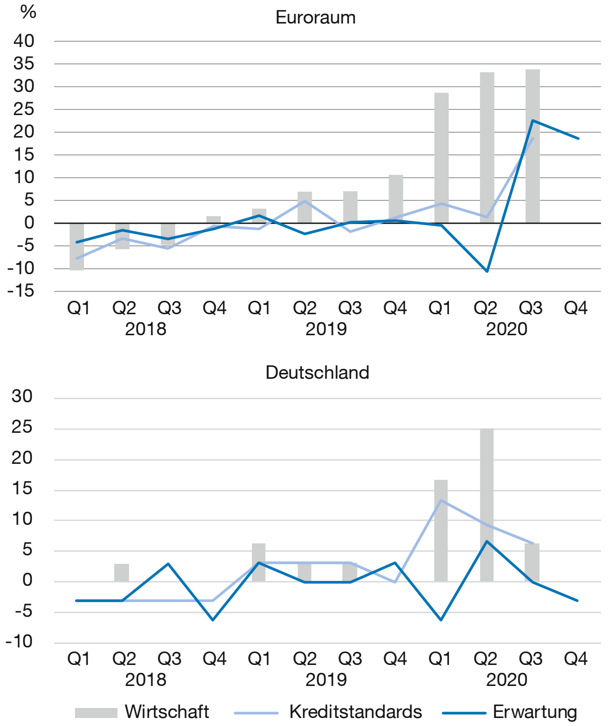

Anmerkung: Anteil der Kreditinstitute mit strengeren Standards im vergangenen Quartal abzüglich Anteil der Institute mit lockereren Standards Positive Werte zeigen eine Verschärfung der Kreditvergabestandards, negative Werte eine Lockerung. Wirtschaft = Auswirkung der wirtschaftlichen Lage (Nettoanteil der Kreditinstitute, die dadurch Standards verschärfen).

Quelle: Europäische Zentralbank, Bank Lending Survey, Kreditangebot.

Entsprechend weisen empirische Mehrländeruntersuchungen darauf hin, dass makroprudenzielle Politik dann schneller und entschlossener agiert, wenn die zuständige Institution, in vielen Ländern ein Finanzstabilitätsausschuss, mit einer starken Governance-Struktur ausgestattet ist. So steigt z.B. die Wahrscheinlichkeit für die Aktivierung antizyklischer Kapitalpuffer, wenn ein Finanzstabilitätsausschuss diese selbst setzen kann, während die Wahrscheinlichkeit für eine Aktivierung mit der Zahl der Mitgliedsinstitutionen sinkt, wahrscheinlich aufgrund von Koordinierungsproblemen und Interessenkonflikten (Edge und Liang, 2020; Gischer et al., 2019). Zudem scheinen antizyklische Kapitalpuffer dann weniger genutzt zu werden, wenn die Kompetenz für ihre Umsetzung bei den (nationalen) Aufsichtsbehörden liegt (Edge und Liang, 2020).

Auch wenn im aktuellen Umfeld durch die Lockerung der Kapitalpuffer keine klassischen Interessenkonflikte zwischen Finanzwirtschaft und Politik vorliegen, spricht strukturell doch einiges dafür, die makroprudenzielle Regulierung so einfach und transparent wie möglich zu organisieren und zu prüfen, ob z.B. der Aufbau des antizyklischen Kapitalpuffers in guten Zeiten hinreichend funktioniert, um das Kernziel seiner Nutzung in Krisenzeiten zu gewährleisten. Denn nur ein adäquater und nicht zu zögerlicher Aufbau im wirtschaftlichen Aufschwung erlaubt größere Spielräume bei der Nutzung von Kapitalpolstern im Abschwung (Jimenez et al., 2017).

## Das Vorgehen der EZB

Um die Nutzung der freigesetzten Eigenkapitalpuffer zu unterstützen, setzt die EZB in erster Linie auf eine Reduktion der Unsicherheit der Kreditinstitute durch drei konkrete Maßnahmen (EZB, 2020). Erstens werden die Institute durch Kommunikation dazu angeregt, die Puffer zu nutzen. Indem die EZB dies öffentlich und nachhaltig tut, trägt sie auch dazu bei, das Signal einer eventuellen Nutzung dieses Puffers als ein positives Signal zu werten. Möglicherweise wirksamer noch sind ex ante Festlegungen zum weiteren Verfahren. In dieser Hinsicht hat die EZB als *zweite* Maßnahme klar kommuniziert, dass die Erleichterungen bei den Eigenkapitalanforderungen wenigstens bis Ende 2022 gelten sollen. *Drittens* hat die EZB die Umstände beschrieben, unter denen es wieder zu einer Normalisierung der Puffer kommen soll.

Es mag im Sinne einer langfristigen Stabilisierung der Erwartungen hilfreich sein, dass solche Überlegungen in Zukunft grundsätzlicher formuliert werden, sodass auch die makroprudenzielle Regulierung stärker den Charakter einer Regelbindung bekommt. Anders als für den Aufbau von Puffern liegen vor allem für die Reduktion von antizyklischen Pufferanforderungen bislang in den meisten Ländern kaum standardisierte Informationen zum Vorgehen der Regulierungsbehörden vor (Arbatli-Saxegaard und Muneer, 2020). Außerdem könnte die Zurückhaltung bei der Aktivierung der antizyklischen Kapitalpuffer, die durch Einflussnahme von Interessengruppen und Koordinierungsfriktionen entsteht, adressiert werden, indem in wirtschaftlich guten Zeiten ein automatischer und früher Aufbau der Pufferanforderungen eingeführt wird, wie z.B. in Dänemark, Norwegen oder Großbritannien.[5] Alternativ könnte ein positives, neutrales Niveau dieses Puffers anvisiert werden. Damit könnte die Nutzung der dann höheren verfügbaren Puffer im Abschwung erleichtert werden.

Eine Ergänzung zur stärkeren Regelbindung könnte darin liegen, die Nutzung von Eigenkapitalpuffern an Bedingungen zu knüpfen. Solche verbindlichen „konditionierten Puffer“ müssten regulär aufgebaut werden, und im Abschwung stehen sie nur zur Verfügung, wenn Bedingungen erfüllt werden, wie etwa eine Vorgabe zum gesamten Kreditvo-

5 So eine Regelbindung müsste über die derzeitige regelgeleitete Komponente beim antizyklischen Kapitalpuffer hinausgehen, denn der derzeit beachtete univariate Zusammenhang zwischen Kredit/BIP-Lücke und Pufferrichtwert ist weder inhaltlich überzeugend noch verbindlich.

lumen. Damit könnte darauf hingewirkt werden, dass die Puffer auch genutzt werden. Allerdings besteht die Gefahr, dass die Handhabung wiederum komplex wird, was nicht wünschenswert ist. Insofern kommt es darauf an, eine tatsächlich leicht umzusetzende Vorgehensweise zu finden.

Mit Blick auf die Kommunikation könnte die Nutzung der Kapitalpuffer gefördert werden, indem (in Anlehnung an die Geldpolitik) mit dem Instrument der Forward Guidance die Transparenz und Planbarkeit erhöht wird. Um die Wirksamkeit der Rücknahme von Kapitalanforderungen zu stärken, ist es auch zielführend, die Aufhebung der Pufferanforderung vollständig der Stärkung der Eigenkapitalbasis zukommen zu lassen. Gegenwärtig sind auch noch Kapitalausschüttungen zulässig.

## Ausblick

Insgesamt scheint es schwierig, die Asymmetrie der Eigenkapitalpuffer im Hinblick auf eine antizyklische Stabilisierung vollkommen aufzuheben. Vielleicht bleibt dies ähnlich wie bei der Geldpolitik, indem die restriktive Wirkung klarer ist als die expansive, weil man im ersten Fall die Entscheidungen der Kreditinstitute direkt restringiert, während im zweiten Fall die Institute einen Freiheitsgrad behalten.

Über die Maßnahmen der EZB hinaus sollten drei Gesichtspunkte diskutiert werden, die alle auf eine einfache makroprudenzielle Regulierung im Abschwung zielen: *Erstens* sollte die makroprudenzielle Regulierung institutionell möglichst wenig komplex organisiert sein, um deren Funktionsfähigkeit zu sichern. *Zweitens* sollte man darüber nachdenken, den regelgebundenen Charakter der makroprudenziellen Politik zu stärken, besonders in guten Zeiten, in denen Kapitalpuffer aufgebaut werden. Dazu gehört *drittens*, dass diese Politik dazu stehen sollte, den Spielraum der Marktakteur:innen im gesamtwirtschaftlichen Interesse zeitweise einschränken zu müssen. Wenn also Kapitalreserven im Sinne einer Stabilisierung der Kreditvergabe oder der Verlustabsorption freigegeben werden, dann scheint es kontraproduktiv, dass die Institute gleichzeitig gegebenenfalls Ausschüttungen vornehmen. Insgesamt wird die zukünftige Ausgestaltung von Eigenkapitalpuffern sicher von den aktuellen Erfahrungen profitieren.
